# Supplementary material for: Nucleolar release of rDNA repeats for repair involves SUMO-mediated untethering by the Cdc48/p97 segregase
Source: Nat Commun. 2021 Aug 13;12:4918. doi: 10.1038/s41467-021-25205-2 (PMC8363623; doi:10.1038/s41467-021-25205-2)
Supplement: Supplementary file 1 — Supplementary Information [file 41467_2021_25205_MOESM1_ESM.pdf]

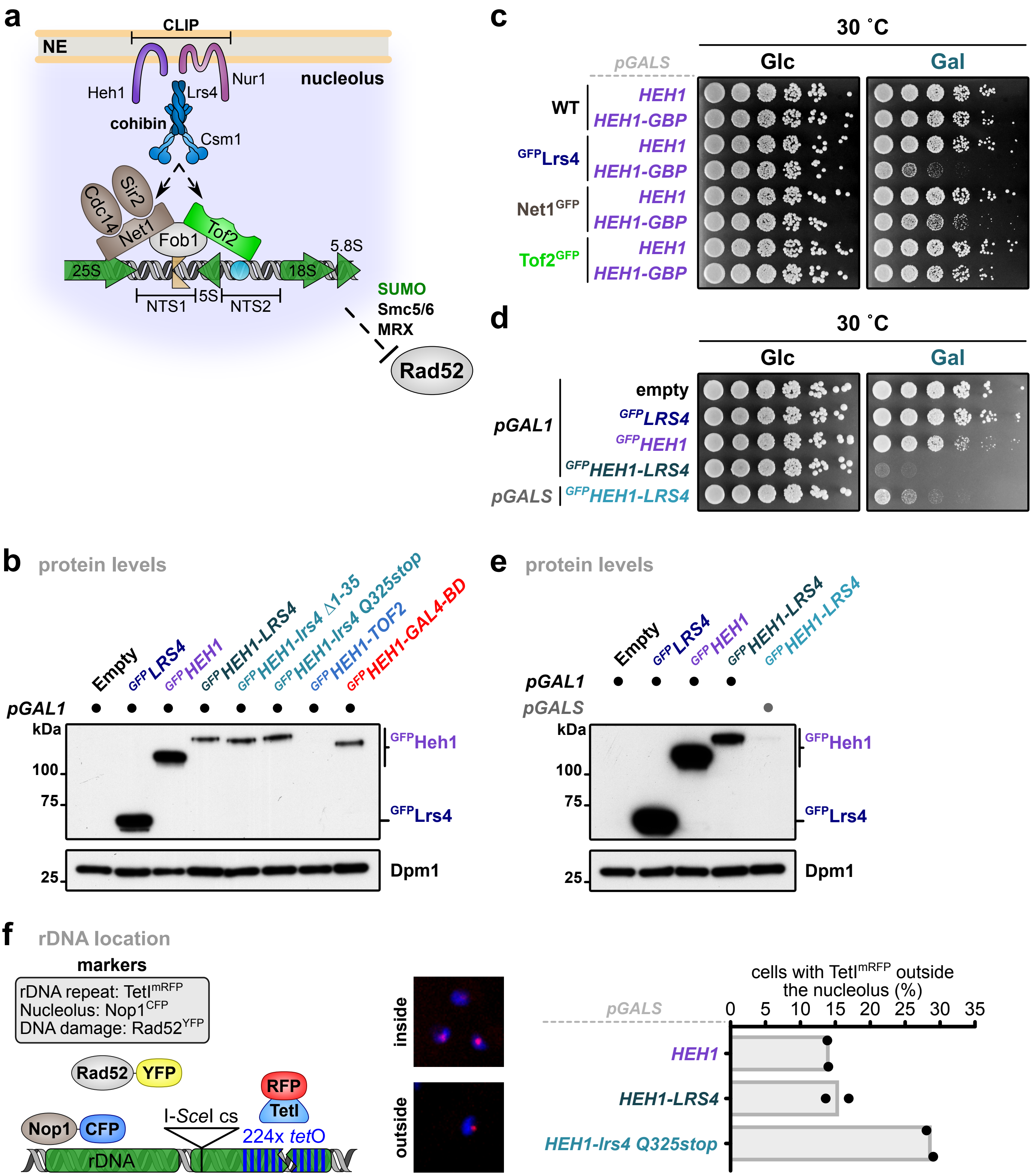

Supplementary Fig. 1 Disruption of rDNA dynamics causes severe growth defects in yeast.
a, Scheme of an rDNA unit bound by the tethering complex in *Saccharomyces cerevisiae*. Each repeat of 9.1 kb presents a near-identical sequence comprising the 35S (subdivided in 5.8S, 18S and 25S) and 5S ribosomal RNA coding genes. Polymerase II silenced regions are known as NTS1 (non-transcribed spacer 1) and NTS2 regions, which are located down- and upstream of the 5S gene, respectively. NTS1 contains the replication fork barrier (RFB), whereas NTS2 contains an autonomous replicating sequence (ARS). The rDNA units are attached to the inner nuclear membrane via interaction between cohibin (Lrs4 and Csm1) and CLIP (Heh1 and Nur1). Cohibin then binds to the NTS1 region of the rDNA repeats through an rDNA-bound protein complex that includes Fob1, Tof2 and the RENT complex (regulator of nucleolar silencing and telophase exit; composed of Cdc14, Net1 and Sir2). SUMO together with the MRX and Smc5/6 complexes exclude Rad52 from the nucleolus. **b**, Immunoblot of WT cells transformed with empty vector or plasmids bearing GFP-tagged *LRS4*, *HEH1* or fusion proteins with the galactose-inducible promoter after induction. Dpm1 served as loading control. **c** and **d**, Five-fold serial dilutions of WT, ^{GFP}Lrs4, Net1^{GFP} and Tof2^{GFP} cells transformed with empty vector or a plasmid bearing *HEH1* fused to GFP-binding protein (GBP) with the weaker *GALS* promoter (**c**); and of WT cells transformed with empty vector or plasmids

Supplementary Fig. 1 (cont.): bearing the indicated GFP-tagged fusion proteins with different galactose-inducible promoters (either *pGAL1* or *pGALS*), as indicated (**d**). Cells were spotted and grown on selective media with 2% glucose (control, Glc) or 2% galactose (induction, Gal) at 30˚C for 3 days. **e**, Immunoblot of the strains used in **d**, after galactose induction. Dpm1 served as loading control. **f**, Left: Scheme of fluorescent markers used to quantify rDNA location. Cells bear a *tetO* array adjacent to an I-*Sce*I endonuclease cut site inserted into an rDNA unit on chromosome XII, which is revealed by $TetI^{mRFP}$ foci. These cells also express $Rad52^{YFP}$ as a marker of the HR machinery. The nucleolus is visualized by a plasmid expressing the nucleolar protein $Nop1^{ECFP}$. Representative images are shown (note that the images are the same as the ones presented in Fig. 2**g**). Scale bar, 2 µm. Right: Percentage of undamaged cells with rDNA repeats localized outside the nucleolus. The different WT strains were transformed with plasmids bearing the indicated GFP-tagged proteins with the *GALS* promoters, and were constantly grown on selective medium with 2% galactose. Repeat location was monitored by the position of $TetI^{mRFP}$ focus relative to the nucleolar mark, and quantification is shown. Data are mean of n = 2 independent biological replicates. For **b** and **e**, the different strains were grown to mid-log phase, fusion proteins were induced by adding 2% galactose for 2 h 30'. Source data are provided as a Source Data file.

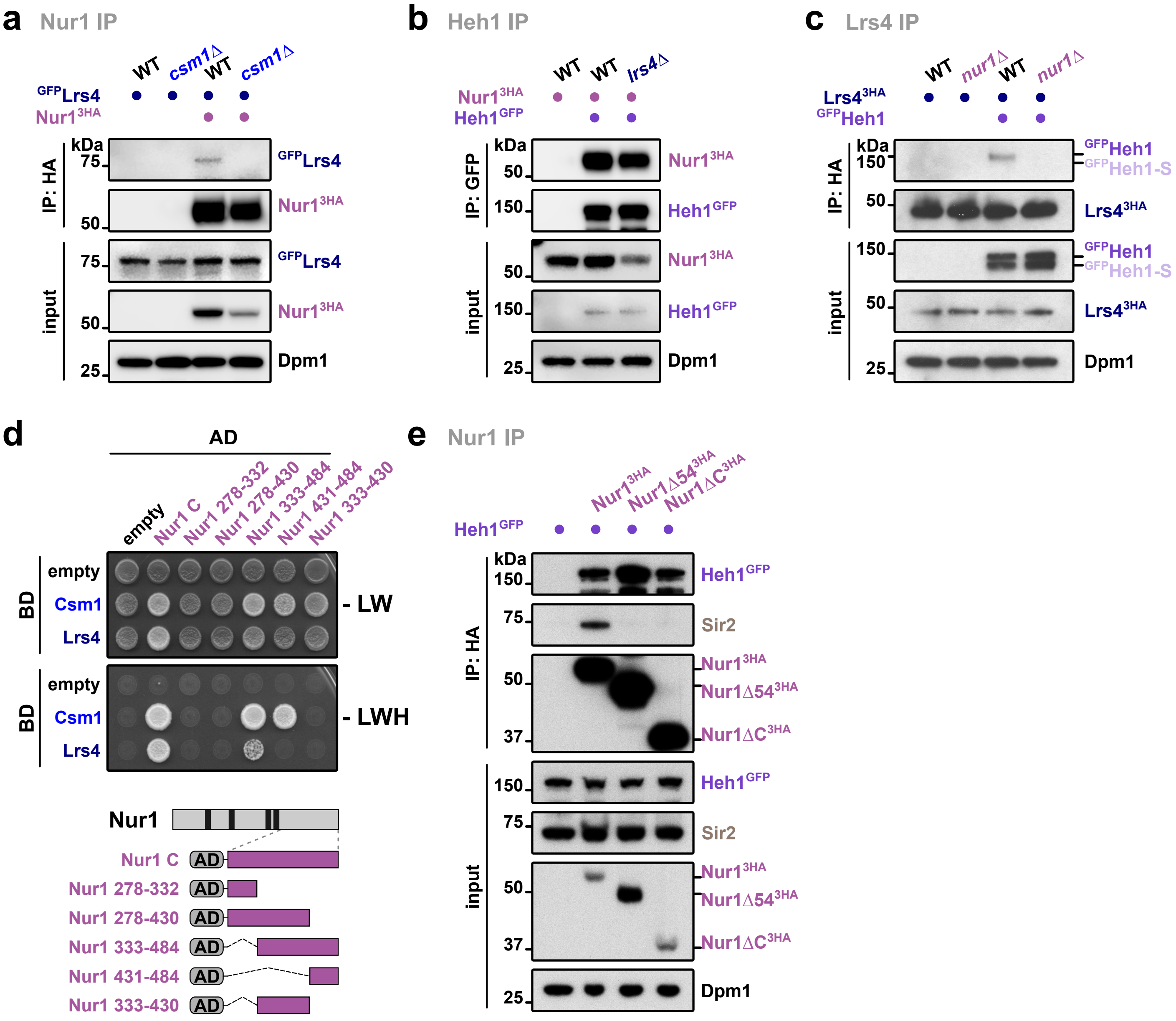

Supplementary Fig. 2 The C-terminal residues of Nur1 are required for CLIP-cohibin complex formation. **a** to **c**, Co-immunoprecipitation of ^{GFP}Lrs4 with Nur1^{3HA} in WT or *csm1Δ* cells (**a**); of Nur1^{3HA} with Heh1^{GFP} in WT or *lrs4Δ* cells (**b**); and of ^{GFP}Heh1 with Lrs4^{3HA} in WT and *nur1Δ* cells (**c**). Due to N-terminal tagging of Heh1, both spliced versions (denoted as Heh1 and Heh1-S) can be detected in **c**. Dpm1 served as loading control. **d**, Y2H analysis of Csm1 and Lrs4 with either Nur1 C or truncated mutants. Fusions with Gal4-activating domain (AD) or Gal4-DNA-binding domain (BD) are indicated. Schematic showing Nur1 truncated constructs. **e**, Co-immunoprecipitation of Heh1^{GFP} and Sir2 with either HA-tagged Nur1 full-length (Nur1^{3HA}), lacking its last 54 residues (Nur1Δ54^{3HA}) or the complete C-terminal domain (Nur1ΔC^{3HA}). Dpm1 served as loading control. For Y2H assays, cells were spotted on control media (-LW) or selective media (-LWH) and grown for 3 days. Source data are provided as a Source Data file.

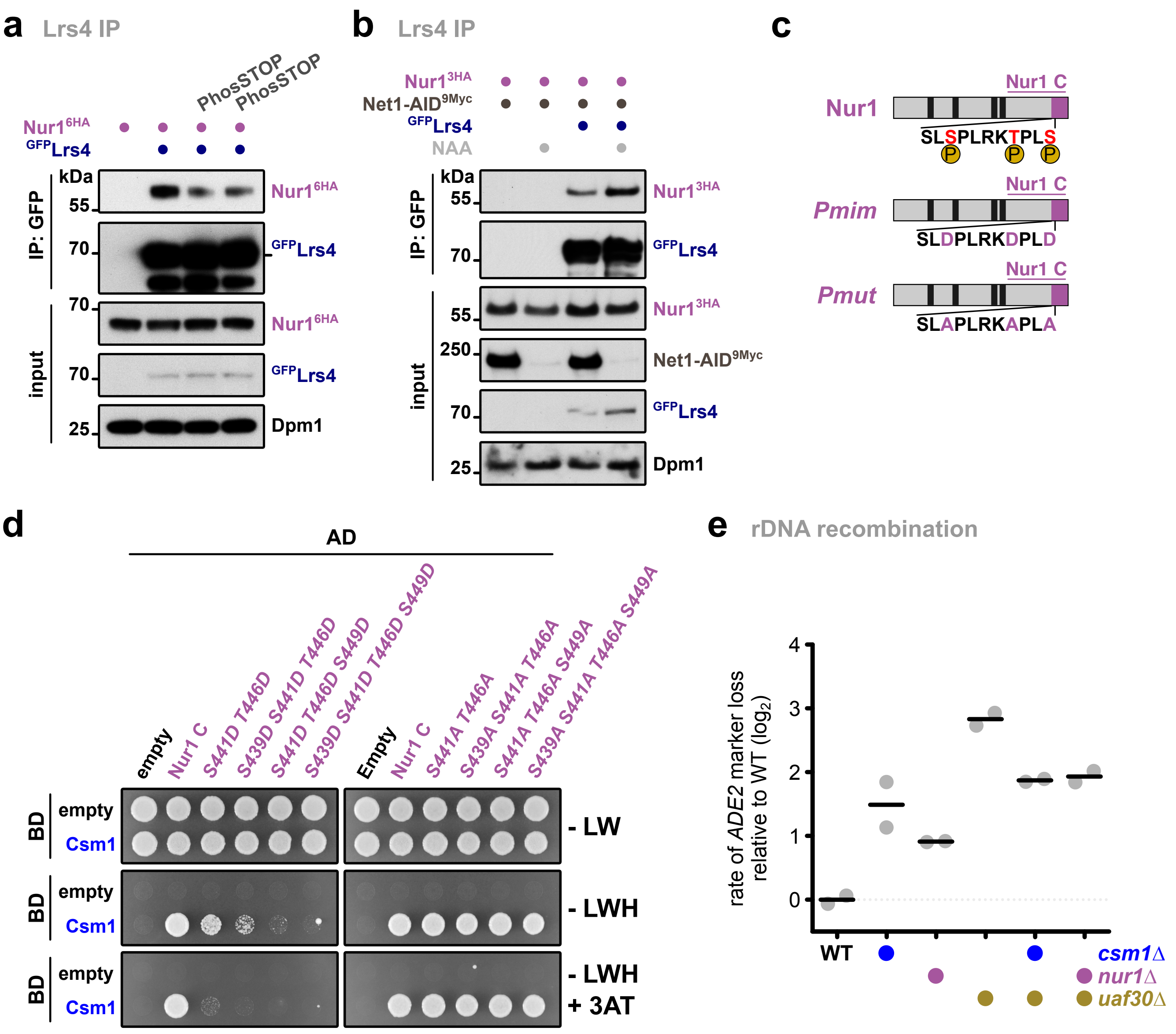

Supplementary Fig. 3 Phosphorylation of C-terminal Nur1 impairs CLIP-cohibin interaction.
a, Co-immunoprecipitation of Nur1^{3HA} with ^{GFP}Lrs4 in WT with impaired global phosphatase activity (PhosSTOP). One untreated and two independently treated samples are shown. Dpm1 served as loading control. **b**, Co-immunoprecipitation of Nur1^{3HA} with ^{GFP}Lrs4 in WT or Net1-depleted cells. Degradation of a C-terminal AID (auxin inducible degron) fusion of Net1 (Net1-AID^{9Myc}) was induced through treatment with 1-naphthaleneacetic acid 1.5 mM (NAA, a synthetic analog of auxin) for 2 h. Dpm1 served as loading control. **c**, Schematic highlighting Nur1 residues important for CLIP-cohibin interaction in red. Amino acids 439 to 449 are depicted. P, phosphorylation. **d**, Y2H analysis of Csm1 (BD) with Nur1 C and phosphomimetic (left) or phosphomutant (right) constructs (AD), as indicated. Cells were spotted on control media (-LW) or selective media (-LWH or -LWH + 3AT 1 mM) and grown for 3 days. **e**, Quantification of rDNA recombination rates in WT, *csm1*Δ, *nur1*Δ, *uaf30*Δ cells, or combination thereof, as measured by loss of an *ADE2* marker inserted into rDNA. The rate of marker loss is calculated as the ratio of half-sectored colonies to the total number of colonies, excluding completely red colonies. Data are mean of n = 2 independent biological replicates, shown in $\log_2$ scale relative to WT. Source data are provided as a Source Data file.

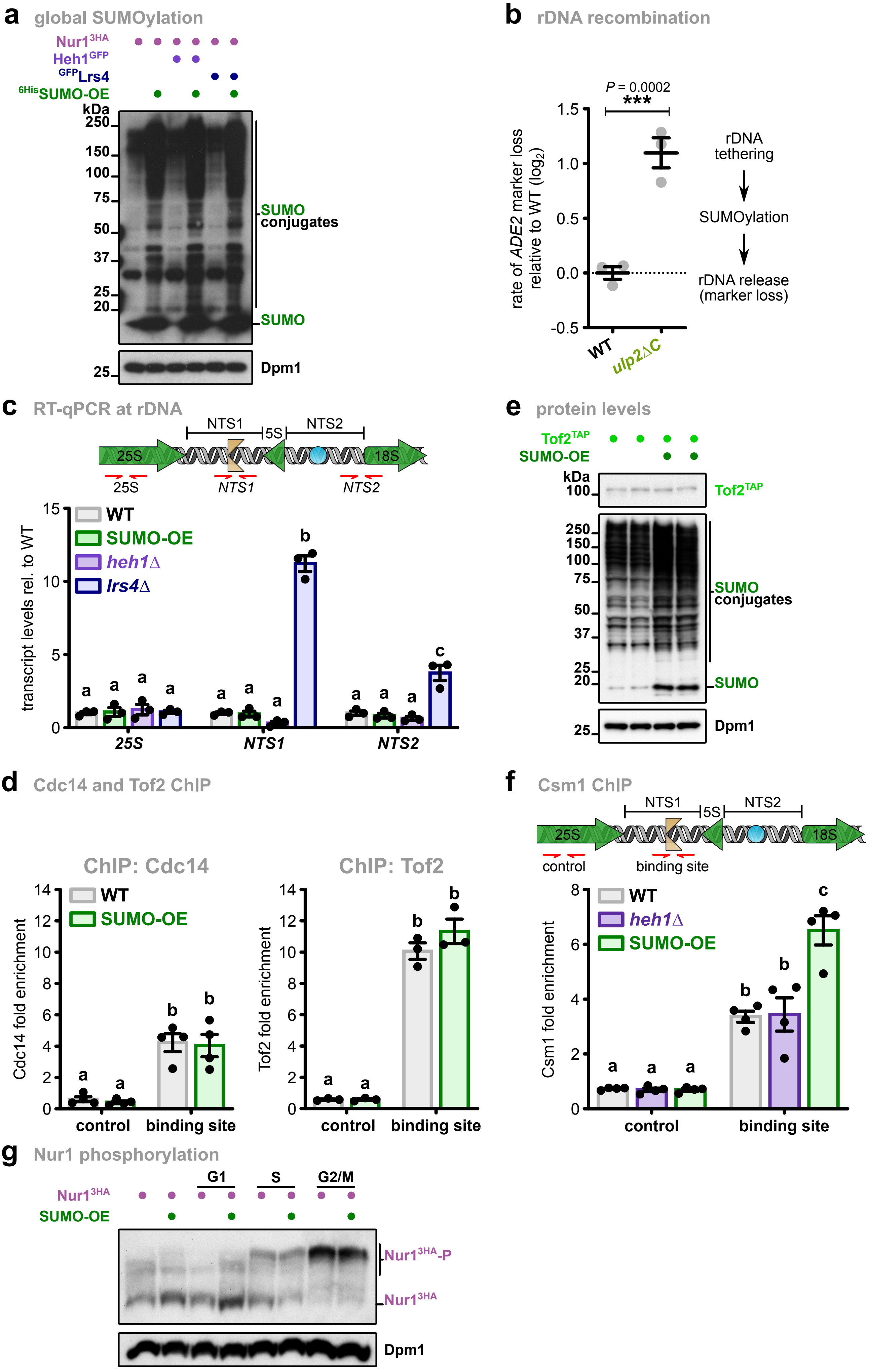
a global SUMOylation
Nur1^3HA
Heh1^GFP
^GFP Lrs4
^6His SUMO-OE
kDa
250
150
100
75
50
37
25
20
25
SUMO conjugates
SUMO
Dpm1
b rDNA recombination
P = 0.0002
***
rate of ADE2 marker loss relative to WT (log2)
1.5
1.0
0.5
0.0
-0.5
WT
ulp2ΔC
rDNA tethering
SUMOylation
rDNA release (marker loss)
c RT-qPCR at rDNA
NTS1
5S
NTS2
25S
18S
25S
NTS1
NTS2
transcript levels rel. to WT
15
10
5
0
WT
SUMO-OE
heh1Δ
lrs4Δ
a
b
c
25S
NTS1
NTS2
e protein levels
Tof2^TAP
SUMO-OE
kDa
100
250
150
100
75
50
37
25
20
25
Tof2^TAP
SUMO conjugates
SUMO
Dpm1
d Cdc14 and Tof2 ChIP
ChIP: Cdc14
ChIP: Tof2
Cdc14 fold enrichment
Tof2 fold enrichment
14
12
10
8
6
4
2
0
WT
SUMO-OE
a
b
control
binding site
f Csm1 ChIP
NTS1
5S
NTS2
25S
18S
control
binding site
Csm1 fold enrichment
8
6
4
2
0
WT
heh1Δ
SUMO-OE
a
b
c
control
binding site
g Nur1 phosphorylation
G1
S
G2/M
Nur1^3HA
SUMO-OE
Nur1^3HA-P
Nur1^3HA
Dpm1

Supplementary Fig. 4 Overexpression of SUMO increases rDNA instability by releasing the repeats from the nucleolus.

a, Immunoblot of SUMO conjugates of cells from Fig. 4**a**, expressing SUMO at endogenous levels or overexpressed from the *ADH1* promoter (^{6His}SUMO-OE). Dpm1 served as loading control. **b**, Rates of rDNA recombination in WT and *ulp2ΔC* cells. The rate of marker loss is calculated as the ratio of half-sectored colonies to the total number of colonies, excluding completely red colonies; data is shown in $\log_2$ scale relative to WT. Statistical analysis was performed using two-tailed Student's t-test from *n* = 3 biological independent experiments. **c**, RT-qPCR analysis of WT, SUMO-OE, *heh1*Δ and *lrs4*Δ strains (*n* = 3). Shown are transcript levels relative to WT after normalization to *ACT1*. Illustration shows a schematic representation of an rDNA unit. Amplified regions are highlighted with red arrows. NTS, non-transcribed spacer; yellow symbol, replication fork block; blue symbol, ARS. **d**, Cdc14^{TAP} (left; *n* = 4) or Tof2^{TAP} (right; *n* = 3) binding to rDNA repeats in cells expressing SUMO at endogenous or overexpressed levels (SUMO-OE), quantified by ChIP-qPCR. SUMO was overexpressed in Cdc14^{TAP} or Tof2^{TAP} strains from vectors with the *GAL1* or the *TEF1* promoter, respectively. The amplified regions are the same as shown in **f**. **e**, Immunoblot of Tof2^{TAP} and SUMO conjugates from strains expressing SUMO at endogenous levels or overexpressed from vectors with the *TEF1* promoter (SUMO-OE). Blots from 2 independent biological clones are shown. Dpm1 served as loading control. **f**, Csm1^{TAP} binding to rDNA repeats in WT, *heh1*Δ and SUMO overexpressing (SUMO-OE) cells, quantified by ChIP-qPCR (*n* = 4). Scheme of an rDNA repeat is shown as in **c**; in red are marked the amplified regions. **g**, Nur1^{3HA} phosphorylation-dependent mobility shifts in cells expressing SUMO at endogenous levels or overexpressed from the *ADH1* promoter (^{6His}SUMO-OE), analyzed using Phos-tag gels (note that molecular weight estimations using markers is not possible with Phos-tag gels). Cells were arrested in G1, S and G2/M by treating the cells with α-factor, HU 100 mM or nocodazole 5 μg/ml for 2 h, respectively. Dpm1 served as loading control. For **b**, **c**, **d** and **f**, data are mean ± SEM of *n* independent biological replicates. For **d** and **f**, the ChIP values are shown as fold enrichment over the average of three rDNA positions, after normalization to input. Statistical analysis was performed using analysis of variance (ANOVA), and different letters denote significant differences with a Tukey's *post hoc* test at $P \leq 0.05$. Source data are provided as a Source Data file.

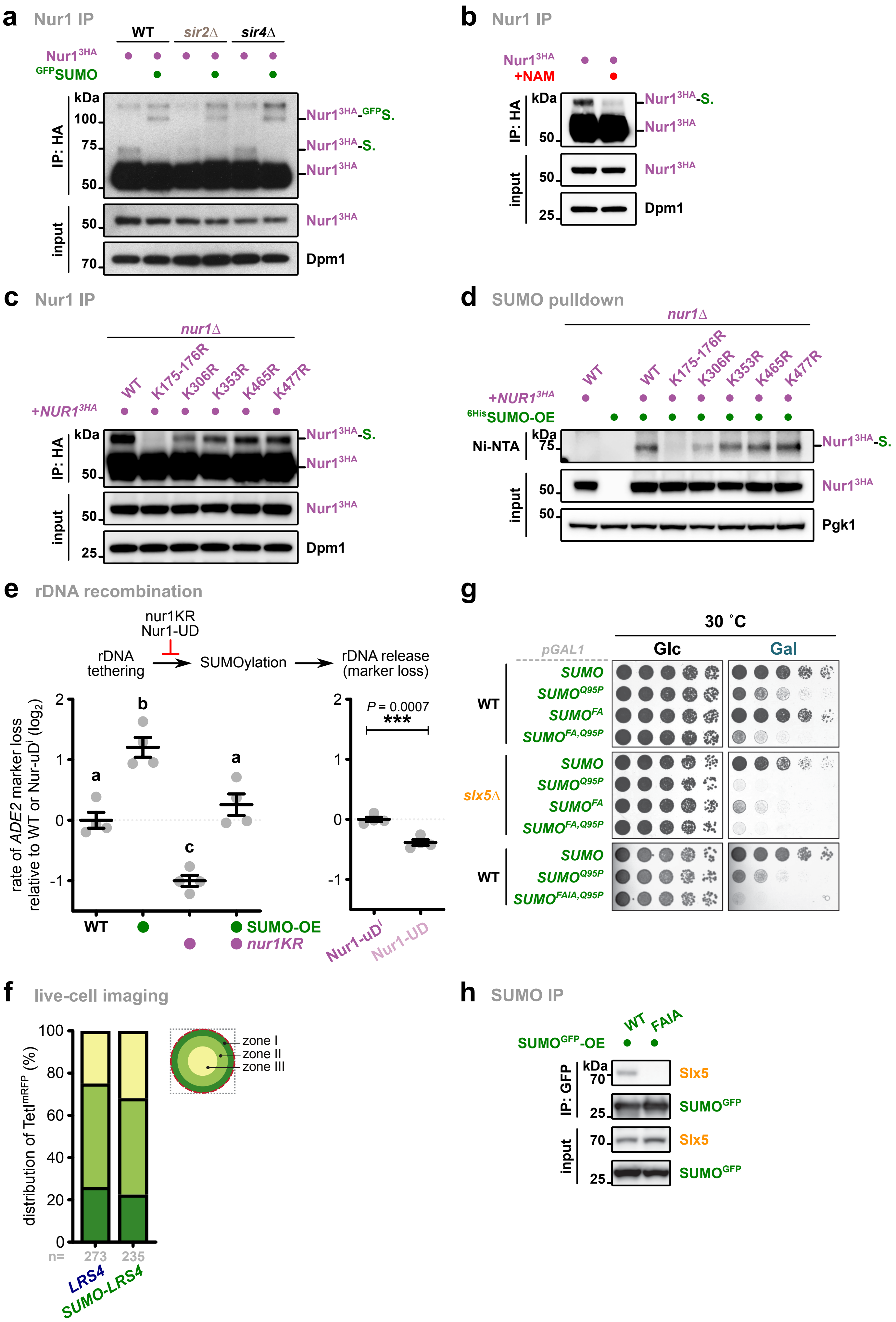
a Nur1 IP
WT
sir2Δ
sir4Δ
Nur1 3HA
GFP SUMO
IP: HA
input
kDa
100
75
50
50
70
Nur1 3HA-GFP S.
Nur1 3HA-S.
Nur1 3HA
Nur1 3HA
Dpm1
b Nur1 IP
Nur1 3HA
+NAM
IP: HA
input
kDa
50
50
25
Nur1 3HA-S.
Nur1 3HA
Nur1 3HA
Dpm1
c Nur1 IP
nur1Δ
WT
K175-176R
K306R
K353R
K465R
K477R
+NUR1 3HA
IP: HA
input
kDa
50
50
25
Nur1 3HA-S.
Nur1 3HA
Nur1 3HA
Dpm1
d SUMO pulldown
nur1Δ
WT
WT
K175-176R
K306R
K353R
K465R
K477R
+NUR1 3HA
6His SUMO-OE
Ni-NTA
input
kDa
75
50
50
Nur1 3HA-S.
Nur1 3HA
Pgk1
e rDNA recombination
nur1KR
Nur1-UD
rDNA tethering
SUMOylation
rDNA release (marker loss)
rate of ADE2 marker loss relative to WT or Nur-uD i (log2)
2
1
0
-1
a
b
c
a
P = 0.0007
***
WT
SUMO-OE
nur1KR
Nur1-uD i
Nur1-UD
f live-cell imaging
distribution of Tetl mRFP (%)
100
80
60
40
20
0
n=
273
235
LRS4
SUMO-LRS4
zone I
zone II
zone III
g
30 °C
pGAL1
Glc
Gal
WT
SUMO
SUMO Q95P
SUMO FA
SUMO FA,Q95P
slx5Δ
SUMO
SUMO Q95P
SUMO FA
SUMO FA,Q95P
WT
SUMO
SUMO Q95P
SUMO FAIA,Q95P
h SUMO IP
WT
FAIA
SUMO GFP-OE
IP: GFP
input
kDa
70
25
70
25
Slx5
SUMO GFP
Slx5
SUMO GFP

Supplementary Fig. 5 SUMOylation of CLIP-cohibin members affects rDNA stability.
a, Immunoprecipitation of Nur1^{3HA} in WT, *sir2*Δ and *sir4*Δ expressing SUMO (endogenous promoter) or ^{GFP}SUMO (*ADH1* promoter). Bands corresponding to Nur1 unmodified or monoSUMOylated are labeled. Dpm1 served as loading control. **b**, Immunoprecipitation of Nur1^{3HA} in WT cells treated with nicotinamide (NAM) 5 mM for 2h. Bands corresponding to Nur1 unmodified or monoSUMOylated are labeled. Dpm1 served as loading control. **c**, Immunoprecipitation of Nur1^{3HA} in *nur1*Δ cells transformed with plasmids bearing *NUR1* or the indicated mutants with the endogenous *NUR1* promoter. Bands corresponding to Nur1 unmodified or monoSUMOylated are labeled. Dpm1 served as loading control. **d**, Denaturing Ni-NTA pulldowns of ^{6His}SUMO conjugates from *nur1*Δ cells transformed with plasmids bearing *NUR1* or the indicated mutants with the endogenous *NUR1* promoter. Pgk1 served as loading control. **e**, Rates of rDNA recombination in WT cells expressing Nur1 or the SUMOylation deficient mutant nur1KR with endogenous or increased levels of SUMO, grown in galactose-containing media (left; *n* = 4); and of strains expressing Nur1 fused to the SUMO-protease domain of Ulp1 (Nur1-UD) or the inactive version (Nur1-uD^{i}), which carries the F474A and C580S mutations (right; *n* = 4). For SUMO overexpression, the different strains were transformed with empty vector or a plasmid bearing SUMO with the *GAL1* promoter. The rate of marker loss is calculated as the ratio of half-sectored colonies to the total number of colonies, excluding completely red colonies, shown in $\log_2$ scale relative to WT (left) or Nur1-uD^{i} (right); data are mean ± SEM of *n* independent biological replicates. Statistical analysis was performed using analysis of variance (ANOVA), and different letters denote significant differences with a Tukey's *post hoc* test at $P \leq 0.05$. **f**, Quantification of TetI^{mRFP} location in *lrs4*Δ cells transformed with plasmids bearing TAP-tagged *LRS4* or *SUMO-LRS4* relative to Nup49^{mars} (nuclear periphery marker), expressed in percentage of cells. n = number of cells counted in 4 biologically independent experiments (scored in strains from Fig. 5**f**). A scheme of a budding yeast nucleus divided in three zones with equal area is shown. These zones are called **I-III** depending on their proximity to the nuclear envelope. **g**, Five-fold serial dilutions of WT or *slx5*Δ cells transformed with plasmids bearing SUMO WT and the indicated SUMO variants with the galactose-inducible promoter *GAL1*. Cells were spotted and grown on selective media with glucose (control, Glc) or galactose (induction, Gal) at 30˚C for 3 days. **h**, Co-immunoprecipitation of Slx5 with either GFP-tagged SUMO WT or a mutant unable to recognize SIMs (FAIA, which harbors the mutations F37A I39A). Source data are provided as a Source Data file.

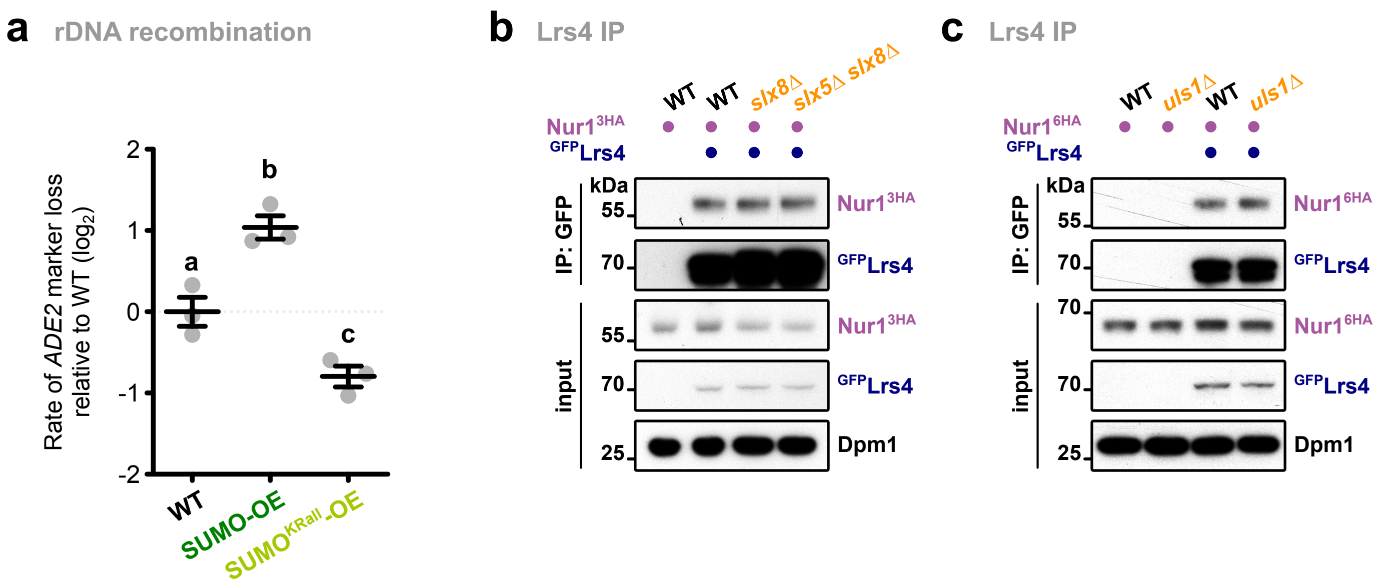

Supplementary Fig. 6 CLIP-cohibin interaction is not affected by the STUbLs

a, Rates of rDNA recombination in WT cells expressing SUMO at endogenous levels or overexpressing either SUMO WT or a mutant unable to form SUMO chains from vectors with the *GAL1* promoter (SUMO-OE or SUMO^{KRall}-OE, respectively), grown in galactose-containing media. The rate of marker loss is calculated as the ratio of half-sectored colonies to the total number of colonies, excluding completely red colonies, shown in log scale relative to WT; data are mean ± SEM of n = 3 biologically independent experiments. Statistical analysis was performed using analysis of variance (ANOVA), and different letters denote significant differences with a Tukey's *post hoc* test at $P \leq 0.05$. **b** and **c**, Co-immunoprecipitation of HA-tagged Nur1 with ^{GFP}Lrs4 in WT, *slx8Δ*, *slx5Δ slx8Δ* (**b**), or *uls1Δ* (**c**) cells, as indicated. Dpm1 served as loading control. Source data are provided as a Source Data file.

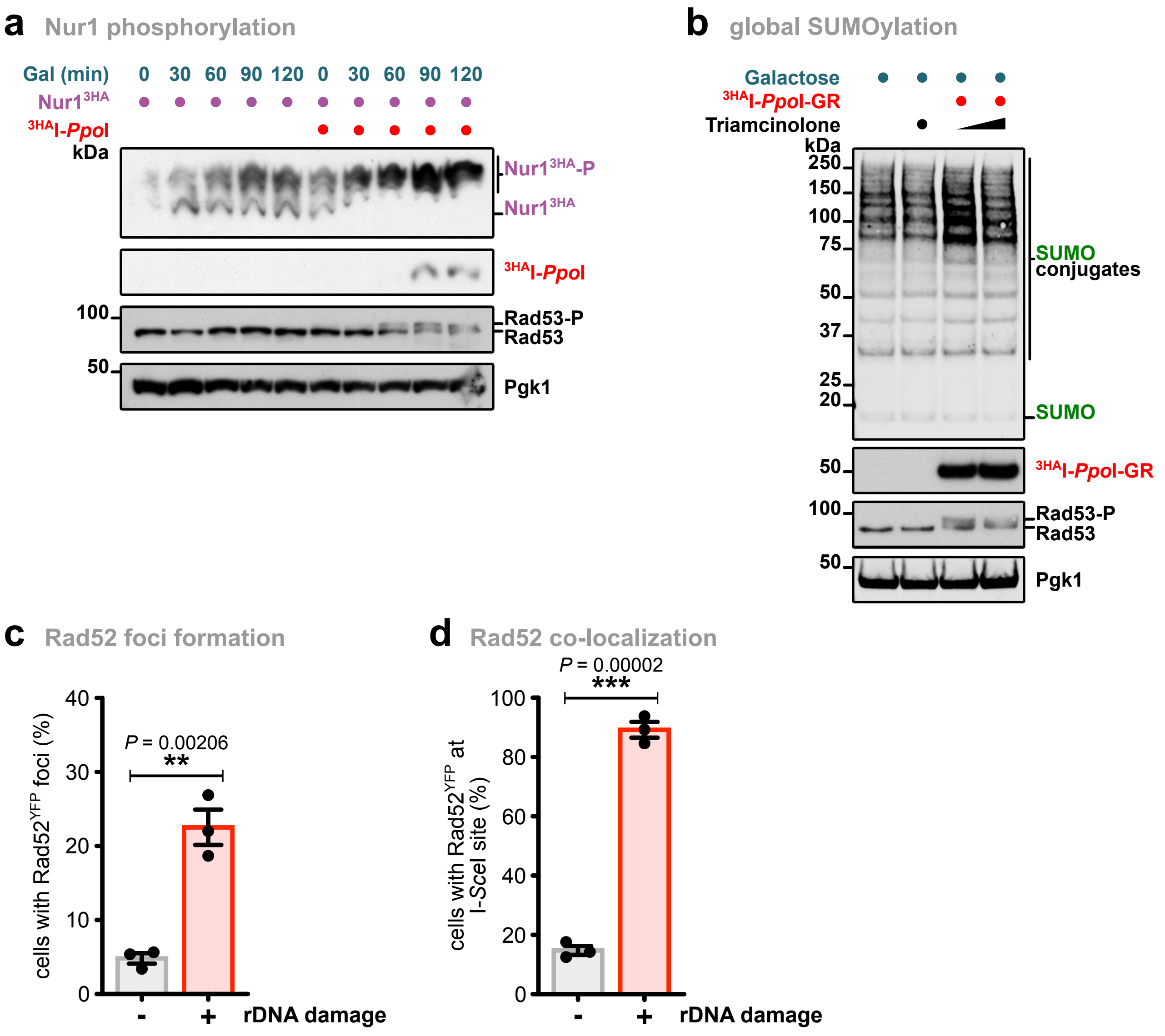

Supplementary Fig. 7 Damage at rDNA promotes release of broken repeats.
a, Nur1^{3HA} phosphorylation-dependent mobility shifts cells in cells with or without rDNA damage, analyzed using Phos-tag gels (note that molecular weight estimations using markers is not possible with Phos-tag gels). ^{3HA}**I-*Ppo*I** with the galactose-inducible promoters *GALL* were integrated at the *LEU2* locus. The different strains were grown to mid-log phase, and the endonuclease was induced by adding 2% galactose for indicated times. **b**, Immunoblot of SUMO conjugates upon rDNA damage. To ensure tight regulation of the endonuclease, **I-*Ppo*I** was fused to the glucocorticoid receptor ligand-binding domain (^{3HA}I-*Ppo*I-GR) and expressed under the galactose-inducible promoter *GAL1* from a centromeric plasmid *YCplac22*. The strains were grown to mid-log phase, and the endonuclease was induced by adding 2% galactose for 3 h. Nuclear location of ^{3HA}I-*Ppo*I-GR was induced by addition of triamcinolone acetonide 7.5 μM and 75 μM. **c**, Percentage of sites with active homologous recombination from WT cells, before (gray) and after (red) DSB induction. Active HR machinery was monitored by Rad52^{YFP} foci formation, scored in strains from Fig. 7**c**. **d**, Percentage of cells with Rad52^{YFP} colocalized with a marked rDNA repeat before (gray) and after (red) DNA damage (I-*Sce*I site) in WT cells. For **a** and **b**, Rad53 phosphorylation served as a marker for DNA damage, while Pgk1 served as loading control. For **c** and **d**, the mean ± SEM of n = 3 independent biological replicates is shown. Statistical analysis was performed using two-tailed Student's t-test, and significant differences are denoted ** ($P \leq 0.005$) and *** ($P \leq 0.001$). Source data are provided as a Source Data file.

Supplementary Table 1. Yeast strains used in this study

Genotype	Source	Identifier
trp1-1 ura3-52 his3Δ200 leu2-3,11 lys2-801	Finley et al., 1987	DF5
DF5, MATα HEH1-L^{EGFP}::HIS3MX6	Capella et al., 2020	MC0064
DF5, MATa HEH1-L^{EGFP}::HIS3MX6 nur1Δ::kanMX6	This study	MC0479
DF5, MATa HEH1-L^{EGFP}::HIS3MX6 CSM1^{TAP}::kanMX6	This study	MC0659
DF5, MATa HEH1-L^{EGFP}::HIS3MX6 nur1Δ::kanMX6 CSM1^{TAP}::kanMX6	This study	MC0184
DF5, MATa LRS4^{3HA}::KlTRP1	This study	MC0628
DF5, MATa LRS4^{3HA}::KlTRP1 nur1Δ::kanMX6	This study	MC0682
DF5, MATα LRS4^{3HA}::KlTRP1 natNT2::pHEH1::^{GFP}HEH1	This study	MC0656
DF5, MATa LRS4^{3HA}::KlTRP1 natNT2::pHEH1::^{GFP}HEH1 nur1Δ::kanMX6	This study	MC0690
DF5, MATα natNT2::pMET25::^{yeGFP}LRS4	This study	MC0073
DF5, MATa natNT2::pMET25::^{yeGFP}LRS4 csm1Δ::hphNT1	This study	MC0089
DF5, MATa natNT2::pMET25::^{yeGFP}LRS4 NUR1^{3HA}::KlTRP1	This study	MC0644
DF5, MATα natNT2::pMET25::^{yeGFP}LRS4 csm1Δ::hphNT1 NUR1^{3HA}::KlTRP1	This study	MC0090
DF5, MATa natNT2::pMET25::^{yeGFP}LRS4 nur1Δ54^{3HA} (1-430aa)::KlTRP1	This study	MC0607
DF5, MATa natNT2::pMET25::^{yeGFP}LRS4 nur1ΔC^{3HA} (1-295aa)::KlTRP1	This study	MC0319
DF5, MATa CSM1^{TAP}::kanMX6	This study	MC0629
DF5, MATa HEH1-L^{EGFP}::HIS3MX6 CSM1^{TAP}::kanMX6 lrs4Δ::hphNT1	This study	MC0761
DF5, MATa HEH1-L^{EGFP} NUR1^{3HA}::KlTRP1	This study	MC0660
DF5, MATa HEH1-L^{EGFP} nur1Δ54^{3HA} (1-430aa)::KlTRP1	This study	MC0606
DF5, MATa HEH1-L^{EGFP} nur1ΔC^{3HA} (1-295aa)::KlTRP1	This study	MC0318
DF5, MATa NET1::AIDx4^{9Myc}::HIS3MX6 URA3::pADH1::OsTIR1^{9Myc} Nur1^{3HA}::KlTRP1	This study	MC0731
DF5, MATa NET1::AIDx4^{9Myc}::HIS3MX6 URA3::pADH1::OsTIR1^{9Myc} Nur1^{3HA}::KlTRP1 natNT2::pMET25::^{yeGFP}LRS4	This study	MC0996
DF5, MATa NUR1^{3HA}::KlTRP1	This study	MC0630
DF5, MATa HEH1-L^{EGFP} NUR1^{3HA}::KlTRP1 lrs4Δ::hphNT1	This study	MC0760
DF5, MATa NUR1^{3HA}::KlTRP1 URA3::pADH1::^{6His}SMT3	This study	MC0723
DF5, MATa HEH1-L^{EGFP} NUR1^{3HA}::KlTRP1 URA3::pADH1::^{6His}SMT3	This study	MC0724
DF5, MATa natNT2::pMET25::^{yeGFP}LRS4 NUR1^{3HA}::KlTRP1 URA3::pADH1::^{6His}SMT3	This study	MC0650
DF5, MATa NUR1^{3HA}::KlTRP1 natNT2::pADH1::^{yeGFP}SMT3	This study	MC0076
DF5, MATa NUR1^{3HA}::KlTRP1 siz1Δ::HIS3MX6	This study	MC0473
DF5, MATa NUR1^{3HA}::KlTRP1 natNT2::pADH1::^{yeGFP}SMT3 siz1Δ::HIS3MX6	This study	MC0474
DF5, MATa NUR1^{3HA}::KlTRP1 siz2Δ::HIS3MX6	This study	MC0475
DF5, MATa NUR1^{3HA}::KlTRP1 natNT2::pADH1::^{yeGFP}SMT3 siz2Δ::HIS3MX6	This study	MC0476
DF5, MATa NUR1^{3HA}::KlTRP1 sir2Δ::HIS3MX6	This study	MC0245
DF5, MATa NUR1^{3HA}::KlTRP1 natNT2::pADH1::^{yeGFP}SMT3 sir2Δ::HIS3MX6	This study	MC0246
DF5, MATa NUR1^{3HA}::KlTRP1 sir4Δ::hphNT1	This study	MC0811

DF5, MATa NUR1^{3HA}::KlTRP1 natNT2::pADH1::^{yeGFP}SMT3 sir4Δ::hphNT1	This study	MC0812
DF5, MATa nur1Δ::kanMX6	This study	MC0634
DF5, MATa nur1Δ::kanMX6 natNT2::pADH1::^{yeGFP}SMT3	This study	MC0077
DF5, MATα URA3::pADH1::^{6His}SMT3	Paasch et al., 2018	MC0435
DF5, MATα heh1Δ::natNT2	Capella et al., 2020	MC0026
DF5, MATα lrs4Δ::hphNT1	This study	MC0429
DF5, MATa CSM1^{TAP}::kanMX6 heh1Δ::natNT2	This study	MC0692
DF5, MATa CSM1^{TAP}::kanMX6 URA3::pADH1::^{6His}SMT3	This study	MC0733
DF5, MATα TOF2^{TAP}::kanMX6	This study	MC0066
DF5, MATa natNT2::pMET25::^{yeGFP}LRS4 NUR1^{3HA}::KlTRP1 slx8Δ::hphNT1	This study	MC0377
DF5, MATa natNT2::pMET25::^{yeGFP}LRS4 NUR1^{3HA}::KlTRP1 slx5Δ::HIS3MX6 slx8Δ::hphNT1	This study	MC0378
MATa ADE2 RAD52^{YFP} RDN25::224xtetO-URA3-I-SceI TetI^{mRFP1}-iYGL119W	Torres-Rosell et al., 2007	ML118-1D
ML118-1D, MATa NUR1^{6HA}::HIS3MX6	This study	MC0857
ML118-1D, MATa nur1 K175-176R^{6HA} (1-430aa)::HIS3MX6	This study	MC0858
ML118-1D, MATa nur1Δ54^{6HA} (1-430aa)::HIS3MX6	This study	MC0859
ML118-1D, MATa nur1ΔC^{6HA} (1-295aa)::HIS3MX6	This study	MC0860
ML118-1D, MATa nur1Δ::kanMX6	This study	MC0678
ML118-1D, MATa SMT3::kanMX6::pADH1::^{6His}SMT3	This study	MC0370
ML118-1D, MATa ufd1ΔSIM (1-355aa)::kanMX6	This study	MC0079
ML118-1D, MATa NUP49^{mars}::hphNT1 lrs4Δ::natNT2	This study	MC1048
trp-901-, leu2-3,112 ura3-53 his3-200 gal4Δ gal80Δ GAL1::HIS GAL2-ADE2 met2::GAL7-lacZ	James et al., 1996	PJ69-7a
MATα ura3-52 his3-200 trp1-901 leu2-3,112 ade2-101 gal4Δ met– gal80Δ URA3::GAL1$_{UAS}$-GAL1$_{TATA}$::LacZ MEL1	Clontech®	Y187
MATa ura3-52 his3-200 trp1-901 leu2-3,112 gal4Δ gal80Δ LYS2::GAL1$_{UAS}$–GAL1$_{TATA::}$HIS3 GAL2$_{UAS}$–GAL2$_{TATA}$::ADE2 URA3::MEL1$_{UAS}$–MEL1$_{TATA::}$AUR1-C MEL1	Clontech®	Y2H Gold
ade2-1 can1-100 his3-11,15 leu2-3,112 trp1-1 ura3-1 RAD5	McDonald et al., 1997	W303 (RAD5)
W303, MATα natNT2::pMET25::^{yeGFP}LRS4	This study	MC0727
W303, MATa NET1^{EGFP}::HIS3MX6	This study	MC0836
W303, MATα TOF2^{EGFP}::HIS3MX6	This study	MC0837
W303, MATa NUR1^{6HA}::HIS3MX6	This study	MC0566
W303, MATa NUR1^{6HA}::HIS3MX6 natNT2::pMET25::^{yeGFP}LRS4	This study	MC0848
W303, MATα NUR1^{6HA}::HIS3MX6 natNT2::pMET25::^{yeGFP}LRS4 cdc14-3	This study	MC0938
W303, MATa NUR1^{6HA}::HIS3MX6 natNT2::pMET25::^{yeGFP}LRS4 cdc14-3	This study	MC0939
W303, MATa NUR1^{6HA}::HIS3MX6 natNT2::pMET25::^{yeGFP}LRS4 ufd1-2::kanMX6	This study	MC0462
W303, MATa NUR1^{6HA}::HIS3MX6 natNT2::pMET25::^{yeGFP}LRS4 ufd1ΔSIM (1-355aa)::kanMX6	This study	MC0478
W303, MATa NUR1^{6HA}::HIS3MX6 uls1Δ::kanMX6	This study	MC0117
W303, MATa NUR1^{6HA}::HIS3MX6 natNT2::pMET25::^{yeGFP}LRS4 uls1Δ::kanMX6	This study	MC0119
W303, MATa HEH1^{EGFP}::HIS3MX6	This study	MC0947
W303, MATa HEH1^{EGFP}::HIS3MX6 LRS4^{TAP}::kanMX6	This study	MC0948

W303, MATa HEH1^{EGFP}::HIS3MX6 LRS4^{TAP}::kanMX6 ChrIII$_{92.5kb}$::natNT2::pGALL::^{3HA}SV40 NLS-I-PPOI	This study	MC0991
W303, MATa HEH1^{EGFP}::HIS3MX6 LRS4^{TAP}::kanMX6 ChrIII$_{92.5kb}$::natNT2::pGAL1::^{3HA}SV40 NLS-I-PPOI	This study	MC0992
W303, MATa NUR1^{6HA}::HIS3MX6 URA3::pGAL1::CDC48::ADHt	This study	MC0163
W303, MATa natNT2::pMET25::^{yeGFP}LRS4 URA3::pGAL1::CDC48::ADHt	This study	MC0164
W303, MATa NUR1^{6HA}::HIS3MX6 natNT2::pMET25::^{yeGFP}LRS4 URA3::pGAL1::CDC48::ADHt	This study	MC0165
W303, MATa NUR1^{6HA}::HIS3MX6 natNT2::pMET25::^{yeGFP}LRS4 URA3::pGAL1::cdc48-6::ADHt	This study	MC0168
W303, MATa NUR1^{6HA}::HIS3MX6 natNT2::pMET25::^{yeGFP}LRS4 URA3::pGAL1::cdc48 E588Q::ADHt	This study	MC0167
W303, MATa CDC14^{TAP}::kanMX6	This study	MC0549
W303, MATa RDN1::ADE2	This study	MC0717
W303, MATa RDN1::ADE2 nur1Δ::kanMX6	This study	MC0669
W303, MATα RDN1::ADE2 csm1Δ::HIS3MX6	This study	MC0953
W303, MATa RDN1::ADE2 uaf30Δ::kanMX6	This study	MC1005
W303, MATα RDN1::ADE2 uaf30Δ::kanMX6 csm1Δ::HIS3MX6	This study	MC1050
W303, MATa RDN1::ADE2 uaf30Δ::kanMX6 nur1Δ::kanMX6	This study	MC1029
W303, MATa RDN1::ADE2 lrs4Δ::hphNT1	This study	MC0641
W303, MATα RDN1::ADE2 ULP2^{6HA}::HIS3MX6	This study	MC0982
W303, MATα RDN1::ADE2 ulp2ΔC^{6HA} (1-781aa)::HIS3MX6	This study	MC0983
W303, MATa RDN1::ADE2 NUR1^{6HA}-ULP1-CD::HIS3MX6	This study	MC1032
W303, MATa RDN1::ADE2 NUR1^{6HA}-ulp1-CDi F474A C580S::HIS3MX6	This study	MC1033
W303, MATa RDN1::ADE2 NUR1^{3HA}::KlTRP1	This study	MC0622
W303, MATa RDN1::ADE2 nur1 K175-176R^{3HA}::KlTRP1	This study	MC0623
W303, MATa RDN1::ADE2 NUR1^{3HA}::KlTRP1 rad52Δ::HIS3MX6	This study	MC0743
W303, MATa RDN1::ADE2 nur1 K175-176R^{3HA}::KlTRP1 rad52Δ::HIS3MX6	This study	MC0744
W303, MATa RDN1::ADE2 NUR1^{6HA}::HIS3MX6	This study	MC0878
W303, MATa RDN1::ADE2 nur1 K175-176R^{6HA}::HIS3MX6	This study	MC0879
W303, MATa RDN1::ADE2 NUR1^{6HA}::HIS3MX6 lrs4Δ::hphNT1	This study	MC0880
W303, MATa RDN1::ADE2 nur1 K175-176R^{6HA}::HIS3MX6 lrs4Δ::hphNT1	This study	MC0881
W303, MATa RDN1::ADE2 ufd1ΔSIM (1-355aa)::kanMX6	This study	MC0160
W303, MATa RDN1::ADE2 ufd1ΔSIM (1-355aa)::kanMX6 lrs4Δ::hphNT1	This study	MC0161
W303, MATa RDN1::ADE2 ufd1ΔSIM (1-355aa)::kanMX6 nur1Δ::natNT2	This study	MC0994
W303, MATa RDN1::ADE2 ctf4Δ::natNT2	This study	MC0773
W303, MATa RDN1::ADE2 ctf4Δ::natNT2 ufd1ΔSIM (1-355aa)::kanMX6	This study	MC0774
W303, MATa RDN1::ADE2 rrm3Δ::natNT2	This study	MC0771
W303, MATa RDN1::ADE2 rrm3Δ::natNT2 ufd1ΔSIM (1-355aa)::kanMX6	This study	MC0772
W303, MATa RDN1::ADE2 NUR1^{6HA}::HIS3MX6 ctf4Δ::natNT2	This study	MC1001

W303, MATa RDN1::ADE2 nur1 K175-176R^{6HA}::HIS3MX6 ctf4Δ::natNT2	This study	MC1002

Supplementary Table 2. Plasmids used in this study.

Point mutations refer to the position of the aminoacid/s (aa) in WT proteins. The specific positions of the truncated constructs are indicated in brackets.

Construct	Source	Identifier
YCplac22	Gietz and Akio, 1988	pMC_0111
YCplac22 pGAL1::HEH1-L-6HA::ADH1t	This study	pMC_0459
YCplac22 pGAL1::HEH1-L-3HA-GBP::ADH1t	This study	pMC_0442
YCplac22 pGALS::HEH1-L-6HA::ADH1t	This study	pMC_0461
YCplac22 pGALS::HEH1-L-3HA-GBP::ADH1t	This study	pMC_0463
YCplac22 pNOP1::NOP1-ECFP	This study	pMC_0480
YCplac22 pGAL1::NUR1::ADH1t	This study	pMC_0595
YCplac22 pGAL1::nur1 S441D T446D S449D::ADH1t	This study	pMC_0599
YCplac22 pGAL1::nur1 S441A T446A S449A::ADH1t	This study	pMC_0600
YCplac111	Gietz and Akio, 1988	pMC_0110
YCplac111 pGAL1::yeGFP-HEH1-L::ADH1t	Capella et al., 2020	pMC_0395
YCplac111 pGAL1::yeGFP-LRS4::ADH1t	This study	pMC_0631
YCplac111 pGAL1::yeGFP-HEH1-L-LRS4::ADH1t	This study	pMC_0443
YCplac111 pGAL1::yeGFP-HEH1-L-lrs4 ∆1-35aa::ADH1t	This study	pMC_0444
YCplac111 pGAL1::yeGFP-HEH1-L-lrs4 Q325Stop::ADH1t	This study	pMC_0445
YCplac111 pGAL1::yeGFP-HEH1-L-TOF2::ADH1t	This study	pMC_0446
YCplac111 pGAL1::yeGFP-HEH1-L-GAL4-BD::ADH1t	This study	pMC_0447
YCplac111 pGALS::yeGFP-HEH1-L-LRS4::ADH1t	This study	pMC_0464
YCplac111 pGALS::yeGFP-HEH1-L-LRS4::ADH1t	This study	pMC_0448
YCplac111 pGALS::yeGFP-HEH1-L-lrs4 Q325Stop::ADH1t	This study	pMC_0465
YCplac111 pNUR1::NUR1::ADH1t	This study	pMC_0565
YCplac111 pNUR1::nur1 S441D T446D S449D::ADH1t	This study	pMC_0570
YCplac111 pNUR1::NUR1-TAP::ADH1t	This study	pMC_0565
YCplac111 pNUR1::nur1 S441D T446D S449D-TAP::ADH1t	This study	pMC_0570
YCplac111 pNUR1::NUR1-3HA	This study	pMC_0545
YCplac111 pNUR1::nur1 K175-176R-3HA	This study	pMC_0546
YCplac111 pNUR1::nur1 K306R-3HA	This study	pMC_0547
YCplac111 pNUR1::nur1 K353R-3HA	This study	pMC_0548
YCplac111 pNUR1::nur1 K465R-3HA	This study	pMC_0549
YCplac111 pNUR1::nur1 K477R-3HA	This study	pMC_0550
YCplac111 pNUR1::smt3 Q95P-NUR1-3HA	This study	pMC_0555
YCplac111 pNUR1::smt3 F37A I39A Q95P-NUR1-3HA	This study	pMC_0556
YCplac111 pLRS4::LRS4-TAP::ADH1t	This study	pMC_0637
YCplac111 pLRS4::smt3 Q95P-LRS4-TAP::ADH1t	This study	pMC_0638
YCplac111 pLRS4::smt3 F37A I39A Q95P-LRS4-TAP::ADH1t	This study	pMC_0639
YCplac111 pNUR1::NUR1-3HA	This study	pMC_0545
YCplac111 pNUR1::smt3 Q95P-NUR1-3HA	This study	pMC_0555
YCplac111 pNUR1::smt3 F37A I39A Q95P-NUR1-3HA	This study	pMC_0546
YCplac111 pGAL1::2xSV40 NLS-HA-I-SceI::ADH1t	This study	pMC_0298
pRS415 pGAL1	Mumberg et al., 1995	pMC_0147
pRS415 pGAL1::3HA-I-PpoI-GR-LBD::CYC1t	This study	pMC_0320

pRS415 pTEF1	This study	pMC_0485
pRS415 pTEF1::SMT3 GG::CYC1t	This study	pMC_0486
pCu415 pGAL1::SMT3 GG::CYC1t	This study	pFA_196
pCu415 pGAL1::smt3 Q95P GG::CYC1t	This study	pFA_261
pCu415 pGAL1::smt3 F37A GG::CYC1t	This study	pFA_264
pCu415 pGAL1::smt3 F37A Q95P GG::CYC1t	This study	pFA_265
pCu415 pGAL1::smt3 F37A I39A GG::CYC1t	This study	pFA_335
pCu415 pGAL1::smt3 F37A I39A Q95P GG::CYC1t	This study	pFA_336
pCu426 pGAL1::smt3 Q95P GG::GFP::CYC1t	This study	pFA_668
pCu426 pGAL1::smt3 F37A I39A Q95P GG::GFP::CYC1t	This study	pFA_669
pCu415 pGAL1::smt3 KRall GG::CYC1t	This study	pFA_321
pGBKT7	Clontech®	pMC_0385
pGBKT7 CSM1	This study	pMC_0477
pGBKT7 LRS4	This study	pMC_0479
pGBKT7 CDC14	This study	pMC_0658
pGBKT7 SMT3 AA	This study	pMC_0273
pGBKT7 smt3 F37A I39A AA	This study	pMC_0275
pGADT7	Clontech®	pMC_0386
pGADT7 HEH1 N (1-454aa)	Capella et al., 2020	pMC_0354
pGADT7 HEH1 C (730-834aa)	Capella et al., 2020	pMC_0355
pGADT7 NUR1 N (1-60aa)	This study	pMC_0505
pGADT7 NUR1 M (167-240aa)	This study	pMC_0507
pGADT7 NUR1 C (278-484aa)	This study	pMC_0504
pGADT7 NUR1 278-332 (278-332aa)	This study	pMC_0524
pGADT7 NUR1 278-430 (278-430aa)	This study	pMC_0523
pGADT7 NUR1 333-484 (333-484aa)	This study	pMC_0525
pGADT7 NUR1 431-484 (431-484aa)	This study	pMC_0527
pGADT7 NUR1 333-430 (333-430aa)	This study	pMC_0526
pGADT7 NUR1 S441D T446D (278-484aa)	This study	pMC_0529
pGADT7 NUR1 S439D S441D T446D (278-484aa)	This study	pMC_0535
pGADT7 NUR1 S441D T446D S449D (278-484aa)	This study	pMC_0537
pGADT7 NUR1 S439D S441D T446D S449D (278-484aa)	This study	pMC_0539
pGADT7 NUR1 S441A T446A (278-484aa)	This study	pMC_0530
pGADT7 NUR1 S439A S441A T446A (278-484aa)	This study	pMC_0536
pGADT7 NUR1 S441A T446A S449A (278-484aa)	This study	pMC_0538
pGADT7 NUR1 S439A S441A T446A S449A (278-484aa)	This study	pMC_0540
pGADT7 SMT3 GG	This study	pMC_0276
pGADT7 SMT3 AA	This study	pMC_0277
pGADT7 CDC48	This study	pMC_0470
pGADT7 NPL4	This study	pMC_0472
pGADT7 UFD1	This study	pMC_0473
pGADT7 ufd1ΔSIM (1-355aa)	This study	pMC_0474

Supplementary Table 3. Set of primers used in this study for qPCR or ChIP experiments.

Primers	Region/Locus	Sequence	Use
MC_636	ACT1 For	GGATTCTGGTATGTTCTAGCGCTTGCACCA	RT-qPCR and genomic DNA normalization
MC_637	ACT1 Rev	AATCTCTCGAGCAATTGGGACCGTGCA	RT-qPCR and genomic DNA normalization
MC_545	I-PpoI cut site For	TGTTGACGCAATGTGATTTCTGCCC	Genomic qPCR (DSB efficiency)
MC_546	I-PpoI cut site Rev	GTAGATAGGGACAGTGGGAATCTCG	Genomic qPCR (DSB efficiency)
MC_699	RDN1 #9 For	CTAGCGAAACCACAGCCAAG	ChIP normalization and control
MC_700	RDN1 #9 Rev	AATGTCTTCAACCCGGATCA	ChIP normalization and control
MC_701	RDN1 #12 For	TAATTGGTTTTTGCGGCTGT	ChIP normalization and RT-qPCR (control)
MC_702	RDN1 #12 Rev	ATGATTTATCCCCACGCAAA	ChIP normalization and RT-qPCR (control)
MC_387	RDN1 #15.1 For	TCACAAAGCTTCCCGAGCGTG	ChIP (binding site) and RT-qPCR (NTS1)
MC_388	RDN1 #15.1 Rev	TCATATCAAAGGCATGTCCTG	ChIP (binding site) and RT-qPCR (NTS1)
MC_393	RDN1 #23 For	GGGAGGTACTTCATGCGAAA	RT-qPCR (NTS2)
MC_394	RDN1 #23 Rev	AAGATGCCCACGATGAGACT	RT-qPCR (NTS2)
MC_711	RDN1 #25 For	GGCAGCAGAGAGACCTGAAA	ChIP normalization and genomic qPCR (rDNA copy number)
MC_712	RDN1 #25 Rev	GAGCCATTCGCAGTTTCACT	ChIP normalization and genomic qPCR (rDNA copy number)

References:

Finley, D., Özkaynak, E. & Varshavsky, A. The yeast polyubiquitin gene is essential for resistance to high temperatures, starvation, and other stresses. *Cell* **48**, 1035–1046 (1987).

Capella, M., Martín Caballero, L., Pfander, B., Braun, S. & Jentsch, S. ESCRT recruitment by the S. cerevisiae inner nuclear membrane protein Heh1 is regulated by Hub1-mediated alternative splicing. *J. Cell Sci.* **133**, jcs250688 (2020).

Paasch, F., Den Brave, F., Psakhye, I., Pfander, B. & Jentsch, S. Failed mitochondrial import and impaired proteostasis trigger SUMOylation of mitochondrial proteins. *J. Biol. Chem.* **293**, 599–609 (2018).

Torres-Rosell, J. *et al.* The Smc5-Smc6 complex and SUMO modification of Rad52 regulates recombinational repair at the ribosomal gene locus. *Nat. Cell Biol.* **9**, 923–931 (2007).

James, P., Halladay, J. & Craig, E. A. Genomic Libraries and a Host Strain Designed for Highly Efficient Two-Hybrid Selection in Yeast. *Genetics* **144**, 1425–1436 (1996).

McDonald, J. P., Levine, A. S. & Woodgate, R. The Saccharomyces cerevisiae RAD30 gene, a homologue of Escherichia coli dinB and umuC, is DNA damage inducible and functions in a novel error-free postreplication repair mechanism. *Genetics* **147**, 1557–1568 (1997).

Gietz, R. D. & Akio, S. New yeast-Escherichia coli shuttle vectors constructed with in vitro mutagenized yeast genes lacking six-base pair restriction sites. *Gene* **74**, 527–534 (1988).

Mumberg, D., Müller, R. & Funk, M. Yeast vectors for the controlled expression of heterologous proteins in different genetic backgrounds. *Gene* **156**, 119–122 (1995).
