# Supplementary material for: Nucleolar release of rDNA repeats for repair involves SUMO-mediated untethering by the Cdc48/p97 segregase
Source: Nat Commun. 2021 Aug 13;12:4918. doi: 10.1038/s41467-021-25205-2 (PMC8363623; doi:10.1038/s41467-021-25205-2)
Supplement: Supplementary file 3 — Reporting Summary [file 41467_2021_25205_MOESM3_ESM.pdf]

nature research

Corresponding author(s): Dr. Sigurd Braun

Last updated by author(s): Jul 9, 2021

Reporting Summary

Nature Research wishes to improve the reproducibility of the work that we publish. This form provides structure for consistency and transparency in reporting. For further information on Nature Research policies, see our Editorial Policies and the Editorial Policy Checklist.

Statistics

For all statistical analyses, confirm that the following items are present in the figure legend, table legend, main text, or Methods section.

n/a	Confirmed	
☐	☒	The exact sample size (n) for each experimental group/condition, given as a discrete number and unit of measurement
☐	☒	A statement on whether measurements were taken from distinct samples or whether the same sample was measured repeatedly
☐	☒	The statistical test(s) used AND whether they are one- or two-sided<br>*Only common tests should be described solely by name; describe more complex techniques in the Methods section.*
☒	☐	A description of all covariates tested
☐	☒	A description of any assumptions or corrections, such as tests of normality and adjustment for multiple comparisons
☐	☒	A full description of the statistical parameters including central tendency (e.g. means) or other basic estimates (e.g. regression coefficient) AND variation (e.g. standard deviation) or associated estimates of uncertainty (e.g. confidence intervals)
☐	☒	For null hypothesis testing, the test statistic (e.g. F, t, r) with confidence intervals, effect sizes, degrees of freedom and P value noted<br>*Give P values as exact values whenever suitable.*
☒	☐	For Bayesian analysis, information on the choice of priors and Markov chain Monte Carlo settings
☒	☐	For hierarchical and complex designs, identification of the appropriate level for tests and full reporting of outcomes
☒	☐	Estimates of effect sizes (e.g. Cohen's d, Pearson's r), indicating how they were calculated

Our web collection on statistics for biologists contains articles on many of the points above.

Software and code

Policy information about availability of computer code

Data collection	Live-cell microscopy images were acquired with a Zeiss AxioObserver Z1 confocal spinning disc microscope with an EMM-CCD camera. Individual ChIP and RT-samples were analyzed by quantitative real-time PCR using QuantStudioTM 3 or QuantStudioTM 5 real-time PCR systems, and processed using Thermo Fisher ConnectTM.
Data analysis	Fiji/ImageJ 2.1.0/1.53c (regularly updated)<br>Graphpad Prism 9.0.1<br>Inkscape 1.0.2<br>Photoshop 12.0.4<br>R (v3.3.3 and v4.0.5)

For manuscripts utilizing custom algorithms or software that are central to the research but not yet described in published literature, software must be made available to editors and reviewers. We strongly encourage code deposition in a community repository (e.g. GitHub). See the Nature Research guidelines for submitting code & software for further information.

Data

Policy information about availability of data

All manuscripts must include a data availability statement. This statement should provide the following information, where applicable:

- Accession codes, unique identifiers, or web links for publicly available datasets
- A list of figures that have associated raw data
- A description of any restrictions on data availability

The data supporting the findings of this study are available within the paper and its supplementary information files.

Field-specific reporting

Please select the one below that is the best fit for your research. If you are not sure, read the appropriate sections before making your selection.

- [x] Life sciences
- [ ] Behavioural & social sciences
- [ ] Ecological, evolutionary & environmental sciences

For a reference copy of the document with all sections, see nature.com/documents/nr-reporting-summary-flat.pdf

Life sciences study design

All studies must disclose on these points even when the disclosure is negative.

Sample size	Sample size of each measurement was based on similar studies and were selected to ensure robust and statistically significant comparisons (Berstein et al., 2013, PMID: 23790361; Ryu and Ahn, 2014; PMID: 25248920; Salvi et al., 2014; PMID: 25073155; Torres-Rosell et al., 2007, PMID: 17643116). No statistical methods were performed to predetermine the sample size.
Data exclusions	No data were excluded from the analysis.
Replication	For all experiments, at least two biological replicates were performed and each replicate was reliably reproduced.
Randomization	For all yeast experiments (ChIP, coIP, IP, Ni-NTA, rDNA marker loss, RT-qPCR, WB, Y2H), cultures were grown under the same conditions and collected randomly without any bias. Microscopy data were generated from randomly selected cells (DIC channel) across several fields before analysis in the respective fluorescence channel.
Blinding	For the analysis, blinded visual scoring of rDNA damage retention in human cells and nucleolar rDNA localization in yeast cells was performed. For all other experiments, no blinding assessment was performed.

Reporting for specific materials, systems and methods

We require information from authors about some types of materials, experimental systems and methods used in many studies. Here, indicate whether each material, system or method listed is relevant to your study. If you are not sure if a list item applies to your research, read the appropriate section before selecting a response.

Materials & experimental systems

n/a	Involved in the study	
[ ]	[x]	Antibodies
[ ]	[x]	Eukaryotic cell lines
[x]	[ ]	Palaeontology and archaeology
[x]	[ ]	Animals and other organisms
[x]	[ ]	Human research participants
[x]	[ ]	Clinical data
[x]	[ ]	Dual use research of concern

Methods

n/a	Involved in the study	
[x]	[ ]	ChIP-seq
[x]	[ ]	Flow cytometry
[x]	[ ]	MRI-based neuroimaging

Antibodies

Antibodies used	- anti-Smt3, Rabbit polyclonal, homemade (Hoege et al., 2002, PMID: 12226657), diluted 1:5,000 for western blot.<br>- anti-Slx5, Rabbit polyclonal, homemade (Höpfler et al., 2019, PMID: 31015336), diluted 1:5,000 for western blot.<br>- anti-Cdc48, Rabbit polyclonal, homemade (Richly et al., 2005, PMID: 15652483), diluted 1:5,000 for western blot.<br>- anti-HA (3F10), Rat monoclonal, Roche Cat# 11867423001, diluted 1:1,000 for western blot.<br>- anti-GFP (B-2), Mouse monoclonal, Santa Cruz Biotechnology Cat# sc-9996, diluted 1:1,000 for western blot.<br>- anti-Dpm1 (5C5A7), Mouse monoclonal, Invitrogen Cat# A-6429, diluted 1:2,000 for western blot.<br>- anti-Pgk1 (22C5D8), Mouse monoclonal, Invitrogen Cat# 459250, diluted 1:5,000 for western blot.<br>- anti-Rad53, Rabbit polyclonal, Abcam Cat# ab104232, diluted 1:1,000 for western blot.<br>- peroxidase anti-peroxidase, Rabbit polyclonal, Sigma Cat# P1291, diluted 1:1,000 for western blot.<br>- anti-Rat IgG/HRP conjugate, Goat polyclonal, Merck Millipore Cat# AP136P, diluted 1:3,000 for western blot.<br>- anti-Mouse IgG/HRP conjugate, Goat polyclonal, BioRad Cat# 170-6516, diluted 1:3,000 for western blot.<br>- anti-Rabbit IgG/HRP conjugate, Goat polyclonal, BioRad Cat# 170-6515, diluted 1:3,000 for western blot.<br>- anti-Nucleophosmin, Rabbit polyclonal, Abcam Cat# ab15440, diluted 1:100 for immunofluorescence.<br>- anti-phospho-H2AX Ser139 (JBW301), Mouse monoclonal, Merck Millipore Cat# 05-636, diluted 1:1,000 for immunofluorescence.<br>- anti-Mouse IgG/Alexa fluor 488, Goat polyclonal, Thermo Fisher Scientific Cat# A11001, diluted 1:5,000 for immunofluorescence.<br>- anti-Rabbit IgG/Alexa fluor 568, Donkey polyclonal, Thermo Fisher Scientific Cat# A10042, diluted 1:5,000 for immunofluorescence.<br>- GFP-Trap Agarose, Chromotek Cat# gta-100, for immunoprecipitation.<br>- anti-HA Affinity Matrix, Roche Cat# 11815016001, for immunoprecipitation.<br>- Rabbit IgG–Agarose, Sigma Cat A2909-5ML, for immunoprecipitation.
Validation	The Smt3, Slx5 and Cdc48 antibodies have been described and validated previously by WB (Hoege et al., 2002, PMID: 12226657, DOI: 10.1038/nature00991; Richly et al., 2005, PMID: 15652483, DOI: 10.1016/j.cell.2004.11.013; Höpfler et al., 2019, PMID: 31015336, DOI: 10.15252/embj.2018100368). All other antibodies are commercially available and were validated by the manufacturers.

Eukaryotic cell lines

Policy information about cell lines

Cell line source(s)	Human cell lines: RPE cells stably expressing I-PpoI were a gift from René H. Medema (Netherlands Cancer Institute).<br>Yeast strains used in this study:<br>DF5: trp1-1 ura3-52 his3Δ200 leu2-3,11 lys2-801<br>MC0064: DF5, MATα HEH1-L-EGFP::HIS3MX6<br>MC0479: DF5, MATa HEH1-L-EGFP::HIS3MX6 nur1Δ::kanMX6<br>MC0659: DF5, MATa HEH1-L-EGFP::HIS3MX6 CSM1-TAP::kanMX6<br>MC0184: DF5, MATa HEH1-L-EGFP::HIS3MX6 nur1Δ::kanMX6 CSM1-TAP::kanMX6<br>MC0628: DF5, MATa LRS4-3HA::KlTRP1<br>MC0682: DF5, MATa LRS4-3HA::KlTRP1 nur1Δ::kanMX6<br>MC0656: DF5, MATα LRS4-3HA::KlTRP1 natNT2::pHEH1::GFP-HEH1<br>MC0690: DF5, MATa LRS4-3HA::KlTRP1 natNT2::pHEH1::GFP-HEH1 nur1Δ::kanMX6<br>MC0073: DF5, MATα natNT2::pMET25::yeGFP-LRS4<br>MC0089: DF5, MATa natNT2::pMET25::yeGFP-LRS4 csm1Δ::hphNT1<br>MC0644: DF5, MATa natNT2::pMET25::yeGFP-LRS4 NUR1-3HA::KlTRP1<br>MC0090: DF5, MATα natNT2::pMET25::yeGFP-LRS4 csm1Δ::hphNT1 NUR1-3HA::KlTRP1<br>MC0607: DF5, MATa natNT2::pMET25::yeGFP-LRS4 nur1Δ54-3HA (1-430aa)::KlTRP1<br>MC0319: DF5, MATa natNT2::pMET25::yeGFP-LRS4 nur1ΔC-3HA (1-295aa)::KlTRP1<br>MC0629: DF5, MATa CSM1-TAP::kanMX6<br>MC0761: DF5, MATa HEH1-L-EGFP::HIS3MX6 CSM1-TAP::kanMX6 lrs4Δ::hphNT1<br>MC0660: DF5, MATa HEH1-L-EGFP NUR1-3HA::KlTRP1<br>MC0606: DF5, MATa HEH1-L-EGFP nur1Δ54-3HA (1-430aa)::KlTRP1<br>MC0318: DF5, MATa HEH1-L-EGFP nur1ΔC-3HA (1-295aa)::KlTRP1<br>MC0731: DF5, MATa NET1::AIDx4-9Myc::HIS3MX6 URA3::pADH1::OsTIR1-9Myc Nur1-3HA::KlTRP1<br>MC0996: DF5, MATa NET1::AIDx4-9Myc::HIS3MX6 URA3::pADH1::OsTIR1-9Myc Nur1-3HA::KlTRP1 natNT2::pMET25::yeGFP-LRS4<br>MC0630: DF5, MATa NUR1-3HA::KlTRP1<br>MC0760: DF5, MATa HEH1-L-EGFP NUR1-3HA::KlTRP1 lrs4Δ::hphNT1<br>MC0723: DF5, MATa NUR1-3HA::KlTRP1 URA3::pADH1::6His-SMT3<br>MC0724: DF5, MATa HEH1-LEGFP NUR1-3HA::KlTRP1 URA3::pADH1::6His-SMT3<br>MC0650: DF5, MATa natNT2::pMET25::yeGFP-LRS4 NUR1-3HA::KlTRP1 URA3::pADH1::6His-SMT3<br>MC0076: DF5, MATa NUR1-3HA::KlTRP1 natNT2::pADH1::yeGFP-SMT3<br>MC0473: DF5, MATa NUR1-3HA::KlTRP1 siz1Δ::HIS3MX6<br>MC0474: DF5, MATa NUR1-3HA::KlTRP1 natNT2::pADH1::yeGFP-SMT3 siz1Δ::HIS3MX6<br>MC0475: DF5, MATa NUR1-3HA::KlTRP1 siz2Δ::HIS3MX6<br>MC0476: DF5, MATa NUR1-3HA::KlTRP1 natNT2::pADH1::yeGFP-SMT3 siz2Δ::HIS3MX6<br>MC0245: DF5, MATa NUR1-3HA::KlTRP1 sir2Δ::HIS3MX6<br>MC0246: DF5, MATa NUR1-3HA::KlTRP1 natNT2::pADH1::yeGFP-SMT3 sir2Δ::HIS3MX6<br>MC0811: DF5, MATa NUR1-3HA::KlTRP1 sir4Δ::hphNT1

MC0812: DF5, MATa NUR1-3HA::KlTRP1 natNT2::pADH1::yeGFP-SMT3 sir4Δ::hphNT1
MC0634: DF5, MATa nur1Δ::kanMX6
MC0077: DF5, MATa nur1Δ::kanMX6 natNT2::pADH1::yeGFP-SMT3
MC0435: DF5, MATα URA3::pADH1::6HisS-MT3
MC0026: DF5, MATα heh1Δ::natNT2
MC0429: DF5, MATα lrs4Δ::hphNT1
MC0692: DF5, MATa CSM1-TAP::kanMX6 heh1Δ::natNT2
MC0733: DF5, MATa CSM1-TAP::kanMX6 URA3::pADH1::6His-SMT3
MC0066: DF5, MATα TOF2-TAP::kanMX6
MC0377: DF5, MATa natNT2::pMET25::yeGFP-LRS4 NUR1-3HA::KlTRP1 slx8Δ::hphNT1
MC0378: DF5, MATa natNT2::pMET25::yeGFP-LRS4 NUR1-3HA::KlTRP1 slx5Δ::HIS3MX6 slx8Δ::hphNT1
ML118-1D: MATa ADE2 RAD52-YFP RDN25::224xtetO-URA3-I-SceI TetI-mRFP1-iYGL119W
MC0857: ML118-1D, MATa NUR1-6HA::HIS3MX6
MC0858: ML118-1D, MATa nur1 K175-176R-6HA (1-430aa)::HIS3MX6
MC0859: ML118-1D, MATa nur1Δ54-6HA (1-430aa)::HIS3MX6
MC0860: ML118-1D, MATa nur1ΔC-6HA (1-295aa)::HIS3MX6
MC0678: ML118-1D, MATa nur1Δ::kanMX6
MC0370: ML118-1D, MATa SMT3::kanMX6::pADH1::6His-SMT3
MC0079: ML118-1D, MATa ufd1ΔSIM (1-355aa)::kanMX6
MC1048: ML118-1D, MATa NUP49-mars::hphNT1 lrs4Δ::natNT2
PJ69-7a: trp-901-, leu2-3,112 ura3-53 his3-200 gal4Δ gal80Δ GAL1::HIS GAL2-ADE2 met2::GAL7-lacZ
Y187: MATα ura3-52 his3-200 trp1-901 leu2-3,112 ade2-101 gal4Δ met– gal80Δ URA3::GAL1UAS-GAL1TATA::LacZ MEL1
Y2H Gold: MATa ura3-52 his3-200 trp1-901 leu2-3,112 gal4Δ gal80Δ LYS2::GAL1UAS–GAL1TATA::HIS3 GAL2UAS–GAL2TATA::ADE2 URA3::MEL1UAS–MEL1TATA::AUR1-C MEL1
W303 (RAD5): ade2-1 can1-100 his3-11,15 leu2-3,112 trp1-1 ura3-1 RAD5
MC0727: W303, MATα natNT2::pMET25::yeGFP-LRS4
MC0836: W303, MATα NET1-EGFP::HIS3MX6
MC0837: W303, MATα TOF2-EGFP::HIS3MX6
MC0566: W303, MATa NUR1-6HA::HIS3MX6
MC0848: W303, MATa NUR1-6HA::HIS3MX6 natNT2::pMET25::yeGFP-LRS4
MC0938: W303, MATα NUR1-6HA::HIS3MX6 natNT2::pMET25::yeGFP-LRS4 cdc14-3
MC0939: W303, MATa NUR1-6HA::HIS3MX6 natNT2::pMET25::yeGFP-LRS4 cdc14-3
MC0462: W303, MATa NUR1-6HA::HIS3MX6 natNT2::pMET25::yeGFP-LRS4 ufd1-2::kanMX6
MC0478: W303, MATa NUR1-6HA::HIS3MX6 natNT2::pMET25::yeGFP-LRS4 ufd1ΔSIM (1-355aa)::kanMX6
MC0117: W303, MATa NUR1-6HA::HIS3MX6 uls1Δ::kanMX6
MC0119: W303, MATa NUR1-6HA::HIS3MX6 natNT2::pMET25::yeGFP-LRS4 uls1Δ::kanMX6
MC0947: W303, MATa HEH1-EGFP::HIS3MX6
MC0948: W303, MATa HEH1-EGFP::HIS3MX6 LRS4-TAP::kanMX6
MC0991: W303, MATa HEH1-EGFP::HIS3MX6 LRS4-TAP::kanMX6 ChrIII92.5kb::natNT2::pGALL::3HA-SV40 NLS-I-PPOI
MC0992: W303, MATa HEH1-EGFP::HIS3MX6 LRS4-TAP::kanMX6 ChrIII92.5kb::natNT2::pGAL1::3HA-SV40 NLS-I-PPOI
MC0163: W303, MATa NUR1-6HA::HIS3MX6 URA3::pGAL1::CDC48::ADHt
MC0164: W303, MATa natNT2::pMET25::yeGFPLRS4 URA3::pGAL1::CDC48::ADHt
MC0165: W303, MATa NUR1-6HA::HIS3MX6 natNT2::pMET25::yeGFP-LRS4 URA3::pGAL1::CDC48::ADHt
MC0168: W303, MATa NUR1-6HA::HIS3MX6 natNT2::pMET25::yeGFP-LRS4 URA3::pGAL1::cdc48-6::ADHt
MC0167: W303, MATa NUR1-6HA::HIS3MX6 natNT2::pMET25::yeGFP-LRS4 URA3::pGAL1::cdc48 E588Q::ADHt
MC0549: W303, MATa CDC14-TAP::kanMX6
MC0717: W303, MATa RDN1::ADE2
MC0669: W303, MATa RDN1::ADE2 nur1Δ::kanMX6
MC0953: W303, MATα RDN1::ADE2 csm1Δ::HIS3MX6
MC1005: W303, MATα RDN1::ADE2 uaf30Δ::kanMX6
MC1050: W303, MATα RDN1::ADE2 uaf30Δ::kanMX6 csm1Δ::HIS3MX6
MC1029: W303, MATa RDN1::ADE2 uaf30Δ::kanMX6 nur1Δ::kanMX6
MC0641: W303, MATa RDN1::ADE2 lrs4Δ::hphNT1
MC0982: W303, MATα RDN1::ADE2 ULP2-6HA::HIS3MX6
MC0983: W303, MATα RDN1::ADE2 ulp2ΔC-6HA (1-781aa)::HIS3MX6
MC1032: W303, MATa RDN1::ADE2 NUR1-6HA-ULP1-CD::HIS3MX6
MC1033: W303, MATa RDN1::ADE2 NUR1-6HA-ulp1-CDi F474A C580S::HIS3MX6
MC0622: W303, MATa RDN1::ADE2 NUR1-3HA::KlTRP1
MC0623: W303, MATa RDN1::ADE2 nur1 K175-176R-3HA::KlTRP1
MC0743: W303, MATa RDN1::ADE2 NUR1-3HA::KlTRP1 rad52Δ::HIS3MX6
MC0744: W303, MATa RDN1::ADE2 nur1 K175-176R-3HA::KlTRP1 rad52Δ::HIS3MX6
MC0878: W303, MATa RDN1::ADE2 NUR1-6HA::HIS3MX6
MC0879: W303, MATa RDN1::ADE2 nur1 K175-176R-6HA::HIS3MX6
MC0880: W303, MATa RDN1::ADE2 NUR1-6HA::HIS3MX6 lrs4Δ::hphNT1
MC0881: W303, MATa RDN1::ADE2 nur1 K175-176R-6HA::HIS3MX6 lrs4Δ::hphNT1
MC0160: W303, MATa RDN1::ADE2 ufd1ΔSIM (1-355aa)::kanMX6
MC0161: W303, MATa RDN1::ADE2 ufd1ΔSIM (1-355aa)::kanMX6 lrs4Δ::hphNT1
MC0994: W303, MATa RDN1::ADE2 ufd1ΔSIM (1-355aa)::kanMX6 nur1Δ::natNT2
MC0773: W303, MATa RDN1::ADE2 ctf4Δ::natNT2

MC0774: W303, MATa RDN1::ADE2 ctf4Δ::natNT2 ufd1ΔSIM (1-355aa)::kanMX6
MC0771: W303, MATa RDN1::ADE2 rrm3Δ::natNT2
MC0772: W303, MATa RDN1::ADE2 rrm3Δ::natNT2 ufd1ΔSIM (1-355aa)::kanMX6
MC1001: W303, MATa RDN1::ADE2 NUR1-6HA::HIS3MX6 ctf4Δ::natNT2
MC1002: W303, MATa RDN1::ADE2 nur1 K175-176R-6HA::HIS3MX6 ctf4Δ::natNT2

Authentication

All strains were generated by transformation and/or using genetic crosses. Strain verification was performed by the presence/absence of selection marker genes and/or by PCR to corroborate the desired genetic modification and/or by western blot to confirm the presence of epitope tags, as appropriate. Human RPE cells were not authenticated.

Mycoplasma contamination

No testing for mycoplasma was required.

Commonly misidentified lines
(See ICLAC register)

No commonly misidentified lines were used.
